# Erratum: Bright nanoscale source of deterministic entangled photon pairs violating Bell’s inequality

**DOI:** 10.1038/s41598-017-07625-7

**Published:** 2017-08-10

**Authors:** Klaus D. Jöns, Lucas Schweickert, Marijn A. M. Versteegh, Dan Dalacu, Philip J. Poole, Angelo Gulinatti, Andrea Giudice, Val Zwiller, Michael E. Reimer

**Affiliations:** 1 Applied Physics Department, Royal Institute of Technology, Albanova University Centre, Roslagstullsbacken 21, 106 91 Stockholm, Sweden; 2 Kavli Institute of Nanoscience, Delft University of Technology, Lorentzweg 1, 2628CJ Delft, The Netherlands; 3 Quantum optics, Quantum Nanophysics and Quantum Information, Faculty of Physics, University of Vienna, Boltzmanngasse 5, 1090 Vienna, Austria; 4 Institute for Quantum Optics and Quantum Information, Austrian Academy of Science, Boltzmanngasse 3, 1090 Vienna, Austria; 5 National Research Council of Canada, Ottawa, K1A 0R6 Canada; 6 Politecnico di Milano, Dipartimento di Elettronica Informazione e Bioingegneria, piazza Leonardo da Vinci 32, 20133 Milano, Italy; 7 Micro Photon Devices, via Stradivari 4, 39100 Bolzano, Italy; 8 Institute for Quantum Computing and Department of Electrical & Computer Engineering, University of Waterloo, Waterloo, N2L 3G1 Canada

*Scientific Reports* **7**:1700; doi:10.1038/s41598-017-01509-6; Article published online 10 May 2017

The original version of this Article contained errors in the Reference list. Reference 36 was incorrectly given as:

“Juska, G., Dimastrodonato, V., Mereni, L. O., Gocalinska, A. & Pelucchi, E. Tuning the optical properties of dilute nitride site controlled quantum dots. *AIP Conference Proceedings* 1566, 447–448 (2013).”

and now reads:

“Juska, G., Dimastrodonato, V., Mereni, L. O., Gocalinska, A. & Pelucchi, E. Towards quantum-dot arrays of entangled photon emitters. *Nat Photon* **7**, 527–531 (2013).”

In addition, Reference 20 was incomplete:

“Zadeh, I. E. *et al*. Deterministic integration of single photon sources in silicon based photonic circuits. *Nano Letters* 0, null (2016).”

now reads:

“Zadeh, I. E. *et al*. Deterministic integration of single photon sources in silicon based photonic circuits. *Nano Letters* **16**, 2289–2294 (2016).”

Additionally, Tables 1 and 2 contained errors in the “Time window (ns)” column, where the values “4.48” and “1.41” were incorrectly given as “0.48” and “0.41”, respectively.

These errors have now been corrected in the HTML and PDF versions of this Article.

